# The effect of acquisition duration on cerebral blood flow‐based resting‐state functional connectivity

**DOI:** 10.1002/hbm.25843

**Published:** 2022-03-26

**Authors:** Yuko Nakamura, Akiko Uematsu, Kazuo Okanoya, Shinsuke Koike

**Affiliations:** ^1^ The UTokyo Center for Integrative Science of Human Behavior (CiSHuB), The University of Tokyo 3‐8‐1, Komaba, Meguro‐ku Tokyo Japan; ^2^ Laboratory for Brain Connectomics Imaging RIKEN Center for Biosystems Dynamics Research Kobe Japan; ^3^ University of Tokyo Institute for Diversity & Adaptation of Human Mind (UTIDAHM) Tokyo Japan; ^4^ International Research Center for Neurointelligence (IRCN) Tokyo Japan; ^5^ Department of Life Sciences Graduate School of Arts and Sciences, The University of Tokyo Tokyo Japan; ^6^ Cognition and Behavior Joint Research Laboratory RIKEN Center for Brain Science Saitama Japan

**Keywords:** acquisition duration, arterial spin labeling, cerebral blood flow, functional magnetic resonance imaging

## Abstract

Resting‐state functional connectivity (rs‐FC) is widely used to examine the functional architecture of the brain, and the blood‐oxygenation‐level‐dependent (BOLD) signal is often utilized for determining rs‐FC. However, the BOLD signal is susceptible to various factors that have less influence on the cerebral blood flow (CBF). Therefore, CBF could comprise an alternative for determining rs‐FC. Since acquisition duration is one of the essential parameters for obtaining reliable rs‐FC, we investigated the effect of acquisition duration on CBF‐based rs‐FC to examine the reliability of CBF‐based rs‐FC. Nineteen participants underwent CBF scanning for a total duration of 50 min. Variance of CBF‐based rs‐FC within the whole brain and 13 large‐scale brain networks at various acquisition durations was compared to that with a 50‐min duration using the Levene's test. Variance of CBF‐based rs‐FC at any durations did not differ from that at a 50‐min duration (*p* > .05). Regarding variance of rs‐FC within each large‐scale brain network, the acquisition duration required to obtain reliable estimates of CBF‐based rs‐FC was shorter than 10 min and varied across large‐scale brain networks. Altogether, an acquisition duration of at least 10 min is required to obtain reliable CBF‐based rs‐FC. These results indicate that CBF‐based resting‐state functional magnetic resonance imaging (rs‐fMRI) with more than 10 min of total acquisition duration could be an alternative method to BOLD‐based rs‐fMRI to obtain reliable rs‐FC.

## 1 INTRODUCTION

Intrinsic functional networks consist of widely distributed brain regions with statistically dependent low‐frequency (<0.1 Hz) fluctuations in activity over time (Buckner, 2010; Deco, Jirsa, & McIntosh, 2011; Fox & Raichle, 2007). The intrinsic functional architecture of the brain can be measured by resting‐state functional magnetic resonance imaging (rs‐fMRI) (Biswal, Yetkin, Haughton, & Hyde, 1995). Due to the experimental simplicity of rs‐fMRI, rs‐fMRI data are easy to collect and share and are often used for biomarker development (Van Essen et al., 2012). Even in children, older adults, and people with cognitive impairment, rs‐fMRI can be performed (Lee, Smyser, & Shimony, 2013); thus, it has become an indispensable technique for neuroimaging studies.

In rs‐fMRI studies, two indices of neural activity are used to measure resting‐state functional connectivity (rs‐FC). One is the blood‐oxygenation‐level‐dependent (BOLD) signal, measured by the gradient‐echo echo‐planar imaging technique, and the other is cerebral blood flow (CBF), measured by the arterial spin labeling (ASL) technique. The BOLD signal reflects changes in deoxyhemoglobin driven by localized changes in brain blood flow and blood oxygenation, which are coupled to the underlying neuronal activity by a process termed neurovascular coupling (Hillman, 2014). CBF is measured using arterial water as an endogenous tracer (Williams, Detre, Leigh, & Koretsky, 1992) and reflects glucose metabolism and neural activity (Borogovac & Asllani, 2012). For CBF measurement, the magnetic labeling of arterial protons is executed upstream from the volume of interest, at the neck vessels, by radiofrequency pulses. Images are acquired after the labeling and inflow period using rapid acquisition techniques. A pair of images is always acquired: a labeled image, in which the blood water magnetization is inverted; and a control image, in which the blood water magnetization is not inverted. The signal difference between the labeled and control images is proportional to the amount of magnetization inverted and delivered to the tissue (Ferré et al., 2013; Haller et al., 2016).

Compared to BOLD‐based rs‐fMRI, the main limitation of CBF‐based rs‐fMRI is its lower signal‐to‐noise ratio (SNR). Furthermore, because the CBF calculation requires (control‐labeled) image subtraction, it has low temporal resolution. Therefore, the BOLD signal is mainly used for rs‐fMRI and has become the standard procedure for assessing rs‐FC. Nonetheless, despite the abovementioned limitations in CBF‐based rs‐fMRI, there are several advantages (Chen, Jann, & Wang, 2015). First, CBF well represents brain metabolism associated with neural activity (Alsop et al., 2015; Cha et al., 2013; Lauritzen, 2001) as well as neurotransmitter activity (Dukart et al., 2018). Moreover, CBF might be able to reflect astrocyte functions, which provide essential metabolic support (Attwell et al., 2010; Kisler, Nelson, Montagne, & Zlokovic, 2017; Marina et al., 2020). Second, because of the processing method used to calculate CBF, the effects of low‐frequency drifts, which is one of the major causes of noises in BOLD‐based rs‐fMRI (Tong, Hocke, & Frederick, 2019), can be minimized, rendering CBF‐based rs‐fMRI ideal for experiments with long measurement duration. Third, CBF is less sensitive to magnetic field inhomogeneity effects and susceptibility‐related signal losses compared with the BOLD signal (Chen et al., 2015). Fourth, CBF is well localized to the capillary beds (Liu & Brown, 2007).

Because of these advantages, CBF‐based rs‐fMRI has been performed to depict intrinsic neural networks in patients with postherpetic neuralgia (Liu et al., 2013), mild cognitive impairment (Li et al., 2019), and chronic fatigue syndrome (Boissoneault et al., 2016). In addition, neural networks depicted by CBF well overlap those derived from the BOLD signal (Jann et al., 2015; Zhang, Huang, & Shah, 2018), and the network strength as measured by CBF is comparable to that with the BOLD signal (Li et al., 2018). Therefore, CBF‐based rs‐fMRI could be an alternative to BOLD‐based rs‐fMRI for studies with long measurement duration or studies aiming to examine brain metabolism.

The acquisition duration is one of the essential parameters for obtaining reliable rs‐FC. Several studies have examined the effect of the acquisition duration on the reliability of BOLD‐based rs‐FC (Anderson, Ferguson, Lopez‐Larson, & Yurgelun‐Todd, 2011; Birn et al., 2013; Elliott et al., 2019; Hacker et al., 2013; Termenon, Jaillard, Delon‐Martin, & Achard, 2016; Van Dijk et al., 2010). These studies have shown that the reliability of rs‐FC depends on the scan length and sample size, including the number of participants and scan volume numbers. In addition, the required acquisition duration for reliable rs‐FC is varied depending on the neural networks evaluated (Elliott et al., 2019). Practically, an acquisition duration of about 20 min could be favorable, although a longer total acquisition duration shows greater reliability (Anderson et al., 2011; Elliott et al., 2019; Hacker et al., 2013).

Unlike the case of BOLD‐based rs‐FC, the effect of the acquisition duration on the reliability of CBF‐based rs‐FC has not been well‐examined. A recent CBF study with a total scan duration of 24 min 30 s showed that a 14‐min acquisition duration is appropriate for obtaining reliable CBF‐based rs‐FC in the estimated default mode network (DMN) (Vallée, Maurel, Corouge, & Barillot, 2020). However, given that increased BOLD‐based rs‐FC reliability has been shown to correspond to increased total acquisition duration (Anderson et al., 2011; Elliott et al., 2019; Hacker et al., 2013), and the required acquisition duration for reliable rs‐FC would be varied across neural networks (Elliott et al., 2019), it is necessary to test the reliability of CBF‐based rs‐FC in various brain networks with longer acquisition durations.

Therefore, in the current study, we conducted an rs‐fMRI experiment to assess the effect of the acquisition duration on CBF‐based rs‐FC. Previous literature showed that 12–30 min scans with 0.7–5 s of repetition time (TR) would be adequate to obtain reliable BOLD‐based rs‐FC (Anderson et al., 2011; Birn et al., 2013; Hacker et al., 2013; Termenon et al., 2016; Van Dijk et al., 2010), and 14 min scan with 3.5‐s TR for reliable CBF‐based rs‐FC (Vallée et al., 2020). Based on these reports, around 270 to 1,200 measurements would be required for BOLD‐based rs‐FC and 130 for CBF‐based rs‐FC. In this study, we thus obtained five 10‐min scans with 2.5‐s TR resulting in 600 measurements in total. Therefore, we assumed that a total duration of 50 min would be sufficient to test the effect of acquisition duration on CBF‐based rs‐FC. Then, we compared variance of rs‐FC of various acquisition durations with that of a 50‐min acquisition duration using time‐series from 90 regions as well as time‐series from 13 large‐scale brain networks. Lastly, rs‐FC variance within each 10‐min scan was calculated to examine the effect of scan repetition.

Given that a previous study with BOLD‐based rs‐FC has showed that the required acquisition duration for reliable rs‐FC is varied across neural networks (Elliott et al., 2019), we hypothesized that the required total acquisition duration for reliable rs‐FC would differ across large‐scale brain networks.

## 2 MATERIALS AND METHODS

### 2.1 Participants

The total CBF scanning duration was 50 min in 19 participants (10‐min scan × 5 runs) (mean age, 21.3 ± 3.3 years; age range, 15.1–29.3 years; 16 males and 3 females).

None of the participants had a history of neurologic or psychiatric disorders. All participants, and their parents if the participant was aged <18 years, provided written informed consent to participate in this study. This study was approved by the ethics committee of the University of Tokyo (Approval no. 20‐297).

### 2.2 MRI acquisition

All the participants were instructed to lie quietly in the scanner and maintain their gaze on a black fixation cross on a white screen during the scan. Every time after the 10‐min scan, the participants were asked whether they kept themselves awake while being scanned to confirm they did not fall asleep, and no participant fell asleep. Therefore, we assumed that they were awake while being scanned.

The scans were acquired on a 3‐T MRI scanner (Magnetom Prisma Fit, Siemens Medical Systems, Munich, Germany) using a 32‐channel head/neck coil.

For CBF scans, the manufacturer's pulsed PICORE (proximal inversion with a control for off‐resonance effects) ASL sequence, with Q2TIPS (quantitative imaging of perfusion using a single subtraction, second version with Thin‐Slice TI1 Periodic Saturation) and 2D‐echo‐planar imaging readout in an ascending order, was used (voxel size: 3.0 × 3.0 × 4 mm^3^; gap: 1 mm; 26 slices; inversion time: 1500 ms; TR/echo time [TE]: 2500/7.5 ms; flip angle: 90°; field of view [FOV]: 252 × 252 mm^2^).

Between the fourth and fifth CBF runs, T1‐weighted high‐resolution anatomical images were acquired with the following parameters: voxel size: 0.8 × 0.8 × 0.8 mm^3^; TR/TE: 2400/2.22 ms; flip angle 8°; FOV: 240 × 256 mm^2^.

### 2.3 Preprocessing MRI data

CBF scans were preprocessed using FSL 6.0.4 (FMRIB, Oxford, UK). Conventional preprocessing was performed as follows for tag and control images together: (a) head motion correction with MCFLIRT (Jenkinson, Bannister, Brady, & Smith, 2002), (b) non‐brain tissue removal with the brain extraction tool (Jenkinson, 2005), (c) spatial smoothing (5‐mm full width at half maximum Gaussian kernel), (d) high‐pass temporal filtering (0.1 Hz, which was 1/4 × TR) to remove slow drifts and potential contamination of BOLD signal (Chuang et al., 2008), and (e) coregistration to structural MRI using boundary‐based registration (Greve & Fischl, 2009). Subsequently, to control for the effect of the head motion, the derivative or root mean square variance over voxels (DVARS) using fsl_motion_outliers (Power, Barnes, Snyder, Schlaggar, & Petersen, 2012) was calculated. Then, to control for the effect of physiological noise, time‐series of cerebral fluid (CSF) and white matter (WM) was extracted from preprocessed ASL images. DVARS, head motion parameters, and time‐series of CSF and WM were regressed out from preprocessed ASL images to control for the effect of head motion and physiological noise. For preprocessed ASL images, linear normalization to the standard MNI space (FLIRT) via the T1‐weighted structural image was applied. Finally, to create simple perfusion images, (control‐label) pairwise subtractions were performed.

### 2.4 Effects of global‐signal regression

Global‐signal regression (GSR) is one of the most controversial preprocessing procedures adopted in BOLD‐based rs‐fMRI preprocessing (Murphy & Fox, 2017). We therefore tested the effect of GSR on CBF‐based rs‐FC. Originally, the GSR was obtained by regressing out the mean time‐series signal across the entire brain mask, including the WM and CSF. However, it has been shown that time‐series signal from the gray matter (GM) provides the largest contribution to the mean time‐series signal (Glasser et al., 2016, 2018; Power, Plitt, Laumann, & Martin, 2017), while WM and CSF contribution beyond partial‐volume effects is negligible (Power et al., 2014). In addition, at the preprocessing, the time‐series from the CSF and WM was regressed out from the ASL time‐series to control for the effect of physiological noise. Therefore, the GSR was performed using the GM time‐series rather than the mean time‐series signal from the whole brain.

To test the effect of global‐signal on CBF‐based rs‐FC, time‐series from the GM volume was extracted and regressed out from preprocessed ASL images to perform GSR (Aquino, Fulcher, Parkes, Sabaroedin, & Fornito, 2020). For preprocessed ASL images with GSR, linear normalization to the standard MNI space via the T1‐weighted structural image was applied. Subsequently, (control‐label) pairwise subtractions were performed to create simple perfusion images.

### 2.5 Resting‐state functional connectivity processing

**FIGURE 1:**
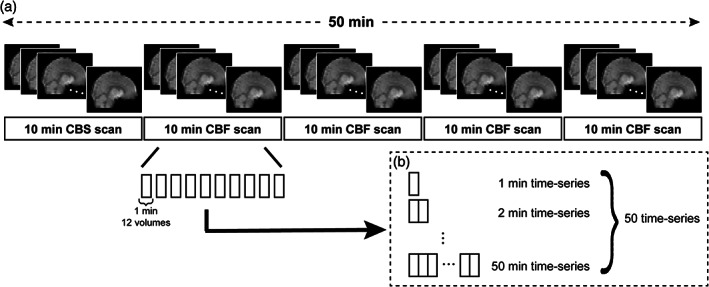
Preparation for time‐series datasets. Each preprocessed 10‐min CBF scan was divided into 1‐min time‐series epochs (a). To create 50 time‐series datasets, 1‐min time‐series epochs were incrementally concatenated with 1‐min time‐series epochs to the first 1‐min time‐series epoch resulting in time‐series datasets ranging 1–50 min (b)

In previous reports, for BOLD‐based rs‐FC, the total scan duration was incremented by 1–5 min each time (e.g., resulting in total scan lengths of 3, 6, and 9 min) to assess the change in the reliability of rs‐FC in relation to the change in total scan length (Anderson et al., 2011; Birn et al., 2013; Elliott et al., 2019; Termenon et al., 2016; Van Dijk et al., 2010). For CBF‐based rs‐FC, the length was progressively incremented by 2 min (Vallée et al., 2020). Therefore, each preprocessed 10‐min CBF scan was divided into 1‐min time‐series epochs to examine the detailed temporal change in the reliability of CBF‐based rs‐FC, and 50 time‐series datasets were created by incrementally concatenating 1‐min time‐series epochs to the first 1‐min time‐series epoch (resulting in time‐series datasets ranging 1–50 min) (Figure 1). CBF time‐series data were then extracted from 90 regions of interest (ROIs), comprising functionally identified brain regions from 14 large‐scale brain networks (Shirer, Ryali, Rykhlevskaia, Menon, & Greicius, 2012). CBF‐based rs‐FC matrices were derived using the Pearson correlation coefficient, and the overall mean CBF‐based rs‐FC was calculated for each time‐series dataset.

Additionally, CBF‐based rs‐FC matrices were calculated for each brain network. Each brain network consisted of 4–10 small regions. Since the primary and secondary visual networks had only four regions in total, we grouped these regions into a single network set as the visual network. CBF time‐series data were extracted from the ROIs within each of the 13 large‐scale brain networks (auditory, basal ganglia, posterior cingulate cortex [PCC]/medial prefrontal cortex [MPFC], primary and secondary visual cortex, language, left dorsolateral prefrontal cortex [DLPFC]/left parietal lobe, sensorimotor, posterior insula, precuneus, right DLPFC/right parietal lobe, insula/dorsal anterior cingulate cortex [dACC], retrosplenial cortex [RSC]/medial temporal lobe [MTL], and intraparietal sulcus [IPS]/frontal eye field [FEF]), and within‐network mean CBF‐based rs‐FC was calculated for each time‐series dataset.

### 2.6 Statistical analysis for overall mean CBF‐based rs‐FC

All statistical analyses were performed using the statistical software package, R version 4.0.3 (The R foundation, Vienna, Austria; https://www.r-project.org/) (R Development Core Team, 2007).

First, assumptions of normality were tested, and the Friedman test was performed to examine the effect of acquisition duration on rs‐FC including rs‐FC as the dependent variable and acquisition duration as the within‐participant variable.

Second, to estimate the reliability of CBF‐based rs‐FC, we compared the variance of overall mean rs‐FC of each time‐series dataset with the variance of overall mean rs‐FC of the 50‐min time‐series dataset using the Levene's test. Since previous findings show that a longer total acquisition duration shows greater reliability (Anderson et al., 2011; Elliott et al., 2019; Hacker et al., 2013), we assumed rs‐FC of the 50‐min time‐series dataset would be the most reliable. We regarded nonsignificance between datasets (uncorrected *p* > .05) as indicating reliable CBF‐based rs‐FC.

Finally, rs‐FC variance was compared across five runs. The assumption of normality was tested using the Shapiro–Wilk's normality test across runs. Since rs‐FC was not normally distributed across runs (all values of *p* < This is "<", not ">.".050), Levene's test was used to evaluate the variance of rs‐FC.

### 2.7 Statistical analysis for mean CBF‐based rs‐FC within each large‐scale brain network

First, assumptions of normality were tested, and the analysis of similarities (ANOSIM) was performed to test differences in rs‐FC across all large‐scale brain networks.

Subsequently, assumptions of normality and sphericity were tested using CBF‐based rs‐FC in each brain network. In brain networks that these assumptions were confirmed, a one‐way repeated‐measures ANOVA was conducted to test the effect of acquisition duration on rs‐FC. In other brain networks, the Friedman test was performed to test the effect of acquisition duration on rs‐FC.

Finally, variance of within‐network mean rs‐FC of each time‐series dataset was compared with variance of the 50‐min time‐series dataset using the Levene's test. We regarded nonsignificance between datasets (uncorrected *p* > .05) as indicating reliable CBF‐based rs‐FC.

### 2.8 Statistical analysis for effects of GSR on overall mean CBF‐based rs‐FC

Since the Shapiro–Wilk's test showed that CBF‐based rs‐FC was not normally distributed at each time‐series epoch, the ANOSIM was performed to test the differences between rs‐FC preprocessed with GSR and rs‐FC without GSR.

## 3 RESULTS

### 3.1 Reliability of overall mean CBF‐based rs‐FC

**FIGURE 2:**
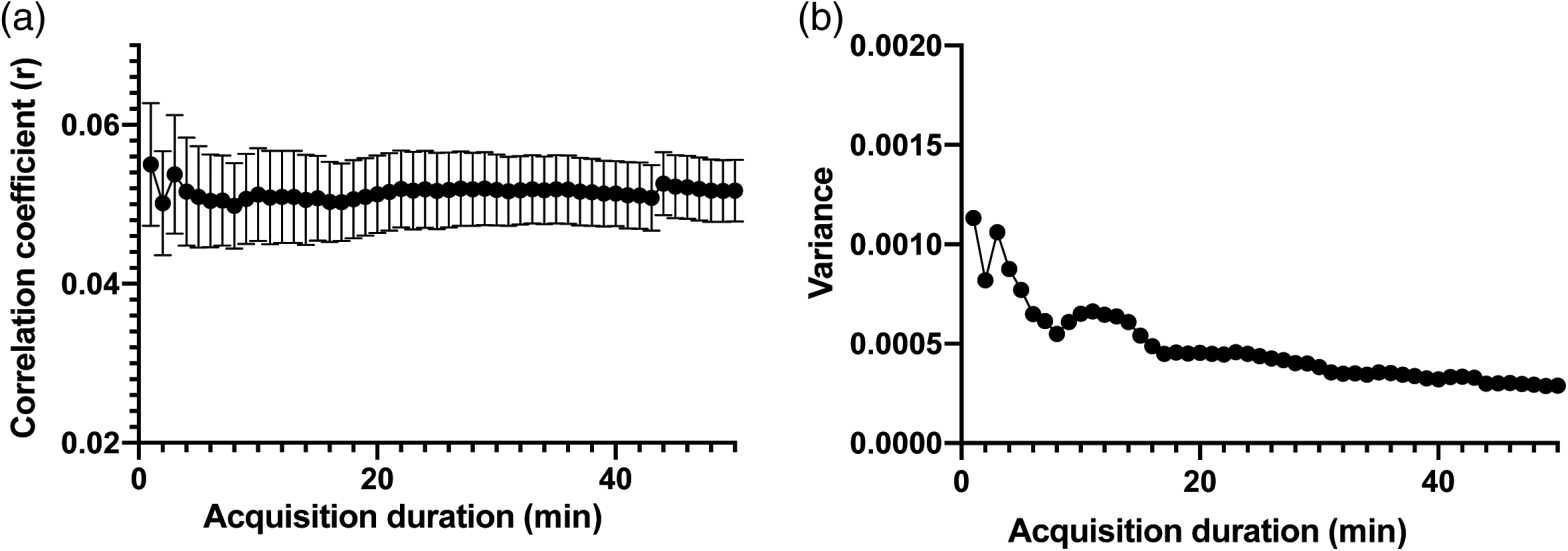
Relationship between the acquisition duration and overall functional connectivity estimate (correlation coefficient) (a) and variance (b). Variance gradually reduced with longer acquisition duration. Error bars depict standard error

The Shapiro–Wilk test showed that rs‐FC was not normally distributed across all time‐series (*p* < .05). Therefore, the Friedman test was performed to test the effect of acquisition duration on CBF‐based rs‐FC. rs‐FC was not statistically significantly different depending on the acquisition duration, *X* ^2^(2) = 40.3, *p* = .0001 (Figure 2a). However, the variance of each time‐series gradually reduced with longer acquisition duration. Therefore, we compared the variance of overall mean rs‐FC of each time‐series dataset with the variance of overall mean rs‐FC of the 50‐min time‐series dataset.

The Levene's test showed that the variance of overall mean rs‐FC with any acquisition duration did not significantly differ from that of the 50‐min time‐series (*p* > .05) (Figure 2b).

### 3.2 The effect of the repetition of scans on variance of CBF‐based rs‐FC

**FIGURE 3:**
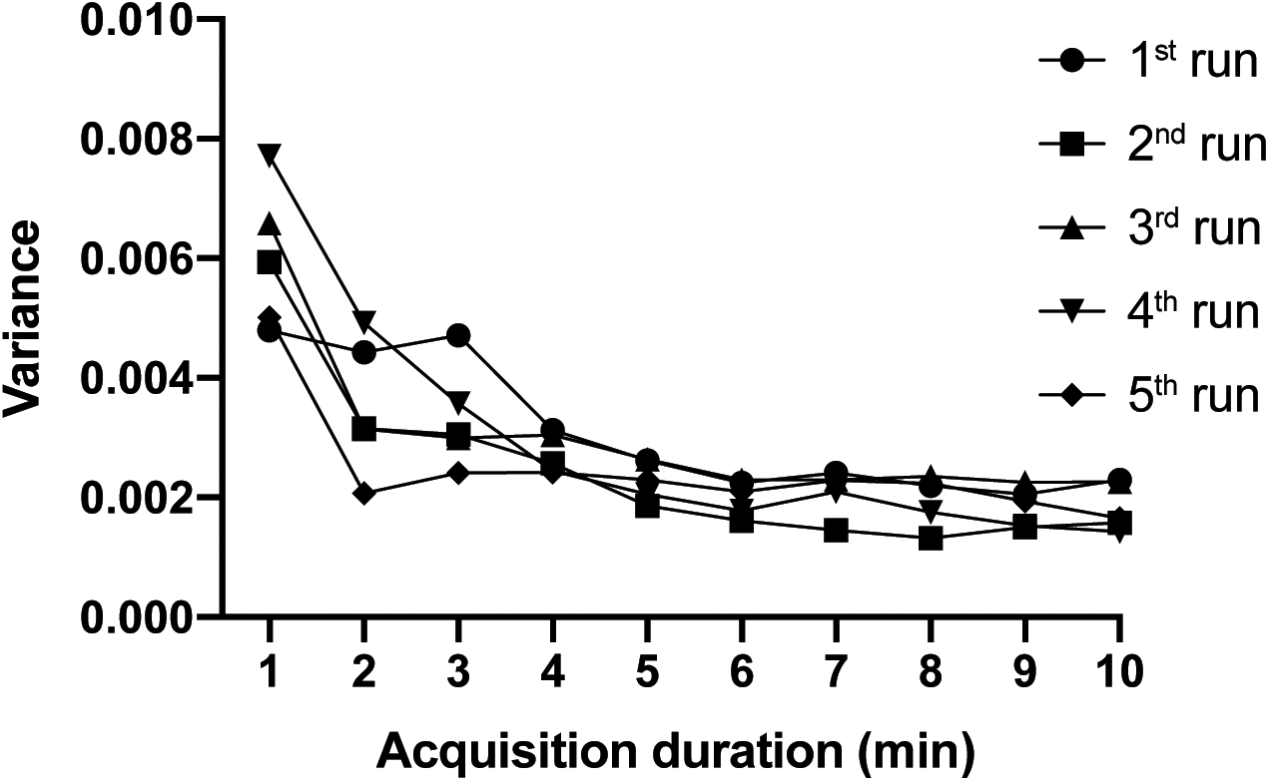
Variance in resting‐state functional connectivity for each run

Levene's test showed a significant difference in rs‐FC variance across the five runs, *F* (4, 945) = 4.407 and *p* = .0016 (Figure 3). As shown in Figure 3, in the third and the fourth run, the variance of shorter time‐series (<5 min) was relatively greater than that of the other runs.

### 3.3 Reliability of mean CBF‐based rs‐FC within each large‐scale network

Since the Shapiro–Wilk's test showed that CBF‐based rs‐FC was not normally distributed at each time‐series, the ANOSIM was performed to test the differences in rs‐FC across all the large‐scale brain networks. The ANOSIM showed that CBF‐based rs‐FC significantly differed across all brain networks (*p* < .001, *R* = .49).

As for each brain network, rs‐FC was normally distributed in the auditory, precuneus, sensorimotor, visual, IPS/FEF, and posterior insula brain networks (*p* > .05), as assessed using the Shapiro–Wilk's test. The Mauchly's test showed that the assumption of sphericity was not violated (*p* > .05) in these brain networks. Therefore, for rs‐FC in these brain networks, a repeated‐measures ANOVA was performed and no significant effect of acquisition duration on rs‐FC was observed [auditory, *F* (49, 882) = 0.234, *p* = 1.00; precuneus, *F* (49, 882) = 0.703, *p* = .94; sensorimotor, *F* (49, 882) = 1.088, *p* = .32; visual, *F* (49, 882) = 0.055, *p* = 1.00; IPS/FEF, *F* (49, 882) = 0.607, *p* = .99; posterior insula, *F* (49, 882) = 0.502, *p* = 1.00]. As for the rest of brain networks, the Shapiro–Wilk's test showed that rs‐FC was not normally distributed in the basal ganglia, left DLPFC/parietal lobe, language, right DLPFC/parietal lobe, insula/dACC, PCC/ MPFC, and RSC/MTL (*p* < .05). The Friedman test showed that there was no significant effect of acquisition duration on rs‐FC [the basal ganglia, *X* ^2^(2) = 40.1, *p* = .81; left DLPFC/parietal lobe, *X* ^2^(2) = 40.7, *p* = .35; language, *X* ^2^(2) = 29.3, *p* = .99; right DLPFC/parietal lobe, *X* ^2^(2) = 45.5, *p* = .62; insula/dACC, *X* ^2^(2) = 25.9, *p* = 1.00; PCC/MPFC, *X* ^2^(2) = 25.9, *p* = 1.00; and RSC/MTL, *X* ^2^(2) = 39.0, *p* = .85].

**FIGURE 4:**
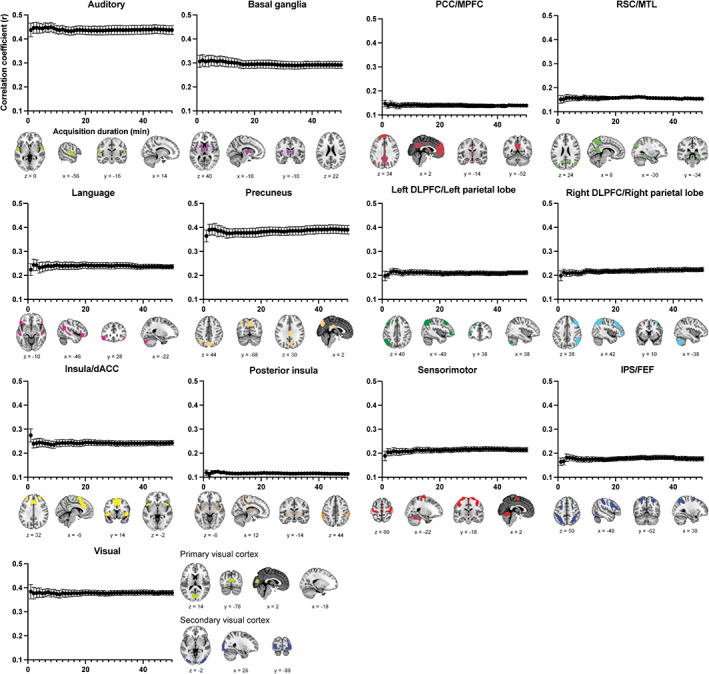
Relationship between the acquisition duration and functional connectivity estimates (correlation coefficient) within each brain network. Error bars depict *SE*. Brain images indicate the ROI for each brain network (adapted from Shirer et al., 2012, with permission from Oxford University Press). The values of *x*, *y*, and *z* indicate the MNI coordinate of brain images. dACC, dorsal anterior cingulate cortex; DLPFC, dorsolateral prefrontal cortex; FEF, frontal eye field; IPS, intraparietal sulcus; MPFC, medial prefrontal cortex; MTL, medial temporal lobe; PCC, posterior cingulate cortex; RSC, retrosplenial cortex

Within‐network mean CBF‐based rs‐FC gradually increased with longer acquisition duration for all the brain networks (Figure 4). Variance of rs‐FC of the 50‐min time‐series did not significantly differ from the variance of rs‐FC at any acquisition duration in the auditory, precuneus, right DLPFC/parietal lobe, and IPS/FEF (*p* > .05). At an acquisition duration of 2 min or longer acquisition duration of the sensorimotor and left DLPFC/parietal lobe, 3 min or longer for the insula/dACC and posterior insula, 4 min or longer for the basal ganglia and RSC/MTL, 6 min or longer for the PCC/MPFC, 9 min or longer for the language, and 10 min or longer for visual brain networks, the variance of within‐network mean CBF‐based rs‐FC did not differ from that of 50‐min time‐series (*p* > .05).

### 3.4 Effects of GSR on CBF‐based rs‐FC

**FIGURE 5:**
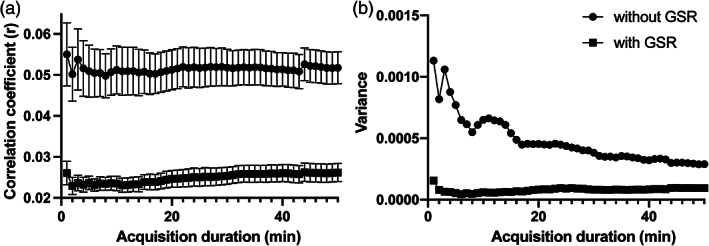
Relationship between the acquisition duration and overall functional connectivity estimate (correlation coefficient) (a) and variance (b) of time‐series with or without global‐signal regression. Error bars depict *SE*. Time‐series with global‐signal regression showed lower functional connectivity and less variability of functional connectivity

The ANOSIM showed that CBF‐based rs‐FC preprocessed with GSR significantly differed from rs‐FC without GSR (*p* < .001, *R* = .46). Although the variance of rs‐FC with GSR decreased compared to the variance of rs‐FC without GSR (Figure 5b), the strength of CBF‐based rs‐FC preprocessed with GSR seemed to be greater than that of rs‐FC without GSR (Figure 5a).

## 4 DISCUSSION

In the present study, we investigated the acquisition duration required to obtain reliable estimates of CBF‐based rs‐FC. Subsequently, we examined the effect of acquisition duration on CBF‐based rs‐FC in each large‐scale brain network. We also tested the effect of GSR on CBF‐based rs‐FC. An acquisition duration did not show a significant effect on the reliability of overall mean CBF‐based rs‐FC. In addition, the acquisition duration required to obtain reliable estimates of CBF‐based rs‐FC varied across large‐scale brain networks. Furthermore, an effect of acquisition duration was not observed in the auditory, precuneus, right DLPFC/parietal lobe, and IPS/FEF networks. Finally, CBF‐based rs‐FC with GSR showed lower strength of rs‐FC compared to that of without GSR.

As shown in Figure 2b, variance of rs‐FC seems to reach a plateau at an acquisition duration of 40 min or longer. Thus, although the variance of rs‐FC with any acquisition durations did not differ from the variance of rs‐FC with 50‐min acquisition duration, 40‐min scan would be favorable in terms of obtaining significant rs‐FC. rs‐fMRI data showed low SNR primarily owing to various physical noise form the scanner (e.g., thermal effect) and physiological noise from the participants (e.g., blood pressure and respiration) (Liu, 2016; Murphy, Birn, & Bandettini, 2013). Such noises of rs‐fMRI data could cause regional variation of rs‐fMRI signal and lead to decreased rs‐FC between different brain regions (Birn et al., 2014; Mueller et al., 2015). To overcome the negative effect of such noises, great amount of data would be needed. Therefore, the variance of CBF‐based rs‐FC decreased with longer acquisition duration similar to the previous studies of BOLD‐based rs‐FC (Anderson et al., 2011; Elliott et al., 2019; Hacker et al., 2013). In addition, longer scans with sparse temporal sampling points (e.g., 12 min, 139 volumes) showed more reliable rs‐FC than shorter scans with the same total scan volumes (e.g., 6 min, 139 volumes) (Birn et al., 2013). Therefore, the scan length itself could have an effect on rs‐FC reliability. Unlike the brain response to stimuli or tasks, the dynamics of spontaneous resting‐state neuronal fluctuations are slow (Buckner, 2010; Deco et al., 2011; Fox & Raichle, 2007; Van Someren, Van Der Werf, Roelfsema, Mansvelder, & Lopes da Silva, 2011). Thus, longer scan lengths could be required to capture the slow changes of resting‐state neuronal fluctuations.

A previous CBF‐based rs‐FC study suggested that a 14‐min acquisition duration would be appropriate for obtaining reliable rs‐FC (Vallée et al., 2020). Using time‐series datasets with various acquisition durations, the authors determined the optimal acquisition duration by examining the overlap in the estimated DMN of each time‐series with a reference DMN from the Multi‐Subjects Dictionary Learning atlas. This evaluation method favors the impact of acquisition duration on the estimation of a specific functional network. However, we utilized a different approach in the present study, exploring the effect of acquisition duration on rs‐FC estimates of various brain networks and on the whole brain. In addition, the ASL sequence in the study by Vallée et al. differed from that in the present study. Furthermore, a difference in TR between Vallée et al. and the current study could be related to differences in required acquisition duration for reliable CBF‐based rs‐FC. rs‐FC reliability depends on sample size, including the number of participants and scan volume numbers (Birn et al., 2013; Termenon et al., 2016). In the current study, the longest acquisition duration to obtain reliable rs‐FC was 10 min for the visual brain network. Since the TR was 2.5 s, the 10‐min duration resulted in 240 volumes, the same number that Vallée et al. suggested for reliable CBF‐based rs‐FC (with TR 3.5 s). These methodological differences could explain the difference in the derived suggested acquisition duration between these studies.

rs‐FC variance of shorter time‐series (<4 min) was greater in the third and fourth runs than variance in the other runs. Previous neuroimaging studies have shown that the patterns in rs‐FC maps can change corresponding to arousal states, such as awake, sleep, or anesthesia state (Banks et al., 2020; Chee & Zhou, 2019; Deco, Hagmann, Hudetz, & Tononi, 2014; Shao et al., 2013). As a previous study with a large sample size (1.147 rs‐fMRI data) reported that 30% of participants do not maintain wakefulness for over 3 min in resting state and resting state could be a dynamic mixture of wakefulness and different sleep stages (Tagliazucchi & Laufs, 2014), unstable arousal levels during rs‐fMRI scans could increase the heterogeneity of intrinsic functional connectivity and result in increased rs‐FC variance. In the current study, after every 10‐min CBF scan, we asked the participants whether they were able to continue further scanning, and only if they were able to undertake scanning, we performed a 10‐min CBF scan until the fourth run in a row. However, even though participants indicated that they were able to undertake scanning, arousal levels could be varied as we continued scanning. After the fourth run, we obtained anatomical images; we then continued with the fifth run, notifying the participants that the fifth run was going to be the last. Given that rs‐FC variance in the fifth scan was decreased from that of the fourth run, arousal levels in the fifth run could have been increased due to the short break to obtain anatomical images or the additional instructions for the fifth run. Overall, as with repeated short scans, arousal levels are varied across participants, even if there are short conversations between scans, resulting in increased rs‐FC variation. These results suggest the need to monitor exact arousal levels during rs‐fMRI scanning.

Across participants, rs‐fMRI data in the same functional network showed substantial similarity (Gratton et al., 2018). In addition, rs‐fMRI data were stable within each cognitive network (Gonzalez‐Castillo et al., 2014). Therefore, within‐network rs‐FC was generally stable, and variance of within‐network rs‐FC seemed smaller than that of overall mean rs‐FC. Additionally, within the auditory, precuneus, right DLPFC/parietal lobe, and IPS/FEF networks, there was no significant effect of acquisition duration on variance of rs‐FC. However, within‐network CBF‐based rs‐FC significantly differed across all brain networks, and brain networks in the MPFC, temporal cortex, and inferior parietal lobes, such as insula and RSC/MTL, showed relatively lower rs‐FC. Since these regions were easily influenced by head motion (Goto et al., 2016) and visual, sensorimotor regions, and insular and auditory cortices were highly influenced by low‐frequency oscillations associated with heart rate and breathing patterns, cardiac pulsatility, breathing motion and head motion (Xifra‐Porxas, Kassinopoulos, & Mitsis, 2021), reliability of CBF‐based rs‐FC in brain networks of these regions would be attenuated due to physical and physiological effects. In addition, in the language and visual networks, relatively longer acquisition durations (9 and 10 min, respectively) were required to obtain reliable rs‐FC. These brain networks are involved in the attentional control (Behrmann, Geng, & Shomstein, 2004; Krall et al., 2015; Posner & Gilbert, 1999; Treue, 2001). During the resting‐state scan, the arousal and attention levels may show inconsistency across and within the participants, leading to increased variance in rs‐FC. Therefore, within these specific brain networks, relatively longer acquisition durations are required to obtain reliable rs‐FC.

GSR is one of the most controversial preprocessing procedures adopted in BOLD‐based rs‐fMRI preprocessing (Murphy & Fox, 2017). We therefore tested the effect of GSR on CBF‐based rs‐FC. GSR is performed with linear regression to remove the whole‐brain average signal from each individual voxel and flatten the time course of it (Murphy & Fox, 2017). Therefore, CBF‐based rs‐FC showed lower variance across all acquisition duration. However, the ANOSIM showed that the strength of CBF‐based rs‐FC without GSR was greater than that of rs‐FC with GSR. Thus, GSR would attenuate the strength of CBF‐based rs‐FC as Elliot and colleagues have reported (Elliott et al., 2019). The impact of GSR on rs‐FC was spatially heterogeneous and the global signal would be associated with neural activation (Xu et al., 2018). Therefore, GSR did not work favorably for CBF‐based rs‐FC values.

To expand the potentials of CBF‐based rs‐FC studies, advantages of the CBF measurement using ASL sequences those that were not used in this study should be considered in future studies to examine the details of brain functions. One of these advantages of CBF measurements is that the absolute CBF values could be calculated using the ASL sequence (Alsop et al., 2015; Buxton, Uludağ, Dubowitz, & Liu, 2004). Absolute CBF measurement is adequate for longitudinal studies (Jain et al., 2012; Ssali et al., 2016) or multisite studies (Mutsaerts et al., 2020) and has been used to depict neural networks (Jann et al., 2013). Another advantage of the CBF measurement that is allowed by recent technical development in ASL is the simultaneous acquisition of CBF‐based MR images and BOLD‐based MR images (Cohen, Nencka, & Wang, 2018; Fernández‐Seara, Rodgers, Englund, & Wehrli, 2016; Schmithorst et al., 2014; Storti, Boscolo Galazzo, Montemezzi, Menegaz, & Pizzini, 2017; Tak, Polimeni, Wang, Yan, & Chen, 2015; Yang, Gu, & Stein, 2004). This technique enables brain activity assessment reflected by BOLD and CBF and has been used to assess the brain response to various tasks (Bangen et al., 2009; Cohen et al., 2018) and functional connectivity (Bangen et al., 2009; Storti et al., 2017; Tak et al., 2015; Zhu, Fang, Hu, Wang, & Rao, 2013). These advanced techniques would be favorable for future CBF‐based rs‐FC studies to examine the effect of development or aging on brain functions using multisite large‐scale MRI data or reveal brain functions using CBF‐ and BOLD‐based measurements.

### 4.1 Limitations

The current study has several limitations. First, in the present study, the scan length was fixed (10 min). Since the effects of low‐frequency drifts on CBF can be smaller than those of BOLD, the effect of scan length on CBF‐based rs‐FC should be tested using various length scans. Second, we performed five 10‐min scans instead of one 50‐min scan to obtain a total duration of 50 min because performing 50‐min scan would not be practical. Between 10‐min scans, we spoke to the participants to confirm whether they were ready for a subsequent 10‐min scan. These short conversations would influence their awake level and modulate CBS‐based rs‐FC. Third, this study included only young adults or adolescents, but not older adults. Given that rs‐FC is often measured in older population because of its simplicity to collect, future studies should examine the effect of acquisition duration on rs‐FC in older populations. The fourth, we have not examined the effect of order of 1‐min time‐series epochs. To create 50 time‐series datasets, 1‐min time‐series epochs could be concatenated in random order. However, given that differences in individual arousal or attention levels across scans could lead to increased differences in variance of rs‐FC across scans, a 1‐min time‐series epoch of one run could be different from that of another run. We thus concatenated 1‐min time‐series epochs in chronological order in this study. In a future study, it would be better to test the effect of the interaction between the scan order and variance on the reliability of rs‐FC.

## 5 CONCLUSIONS

This study examined the required acquisition duration for obtaining reliable CBF‐based rs‐FC, with a total scan length of 50 min, within the whole brain as well as in various large‐scale brain networks. Although the variance of CBF‐based rs‐FC gradually reduced as the acquisition duration increased, there was no significant difference in strength or variance of CBF‐based rs‐FC across various acquisition durations. Furthermore, the variance of within‐network rs‐FC seemed smaller than that of overall mean rs‐FC and 10 min acquisition durations could be adequate to obtain reliable within‐network rs‐FC. These results indicate that CBF‐based rs‐fMRI with more than 10‐min total acquisition duration can be an alternative method to BOLD‐based rs‐fMRI to obtain reliable rs‐FC.

## CONFLICT OF INTEREST

The authors declare no competing financial interests.

## AUTHOR CONTRIBUTIONS

Yuko Nakamura and Shinsuke Koike designed the study. Yuko Nakamura processed, analyzed, and visualized the data and results. Akiko Uematsu preprocessed the fMRI data. All authors reviewed the article, provided critical feedback, and approved the final submission.

## PATIENT CONSENT STATEMENT

No patient was included in the current study. Not applicable.

## PERMISSION TO REPRODUCE MATERIAL FROM OTHER SOURCES

Not applicable. All materials were originally produced for the current study.

## ETHICS STATEMENT

This study was approved by the ethics committee of the University of Tokyo (Approval no. 20‐297).

## Data Availability

The data that support the findings of this study are available from the corresponding author upon reasonable request.
